# The workforce crisis in public psychiatry can be addressed by asking psychiatrists to focus on psychiatry

**DOI:** 10.1177/10398562241286686

**Published:** 2024-09-22

**Authors:** Andrew James Amos

**Affiliations:** Division of Tropical Health and Medicine, College of Medicine and Dentistry, 104397 James Cook University, Townsville, QLD, Australia

To overcome the barriers that prevent health reform, efforts to improve access to mental health care are often framed in the context of high-profile crises. A recent example was the response to the killing of six innocent people at Bondi Beach by a man with long-standing mental illness.^1^

Crisis-driven reform tends to focus on plugging the gaps in resource-constrained systems caused by inadequate funding; the unfair distribution of infrastructure across various populations, demographics, and diagnostic categories; or specific factors affecting potentially vulnerable groups such as people with severe mental illness, veterans, or First Nations people.

While the instinct to respond to crises with promises to increase the number of mental health workers is understandable, it may not be the best way to address the problems facing Australia’s mental health systems. If, as has been argued, too much of the existing mental health workforce targets high prevalence, low acuity conditions like anxiety and sub-clinical low mood,^2^ then simply increasing the number of health staff is unlikely to improve the outcomes of the most disadvantaged.

In addition, it is increasingly recognised that mental health workforce capacity at any point in time is a complex function of many past and present factors including graduate numbers; recruitment/retention based on the perception of mental health as a career and realised working conditions; the intense concentration of health professionals in metropolitan areas; and the funding, priorities, and service demands of the health system itself.^3^

The Productivity Commission report on Mental Health in 2020 remains the most comprehensive analysis of the strategic planning needed to address the growing gap between Australia’s mental health workforce needs and reality.^4^ This report focussed on bridging the gaps using existing models of care and practice. However, it also discussed changes to the scope of practice of clinicians across the health spectrum; from increasing GPs’ mental health skills; through technologies like telehealth and remote psychiatric advice to GPs; to new categories of mental health worker, such as tertiary trained mental health nurses, Peer workers, and First Nations workers.

Although less comprehensive than the Productivity Commission report, another important public policy document is the National Mental Health Workforce Strategy 2022–2032.^5^ In my view, the focus on increasing the number of health workers, both for established roles like psychiatrist and mental health nurse, as well as ramping up new categories like Peer workers and First Nations workers, overshadows the greater potential of reorganising the framework of care within health institutions.

The introduction of Peer workers with lived experience and First Nations workers with shared cultural understanding can improve outcomes for many psychiatric inpatients. However, it is not enough to simply add these and other new professional roles like physicians’ assistants to existing units as optional extras. Their expertise must be leveraged to improve patient outcomes and reduce the workload of other staff, improving the efficiency and effectiveness of the whole team.

The physician’s assistant (PA) is an example of this type of task shifting. This is a type of non-physician clinician who performs some of a doctor’s tasks under their supervision.^6^ While neither the Productivity Commission nor the National Mental Health Workforce Strategy mention the term, Australian public hospital and health services have begun to integrate PAs into practice.^7^ I am not aware of PAs being used in any of Australia’s public mental health services, but the only recent review of PAs in mental health care suggested that 62% of the 139,688 PAs certified to practice in the US evaluated mental health conditions at least weekly,^8^ so it remains a distinct possibility.

There is insufficient evidence to predict whether the addition of PAs to public mental health services is likely to reduce the workload of other clinicians or improve patient care. However, the theory that PAs can reduce physician workload and allow them to focus on higher-level aspects of care that require psychiatric expertise should be more broadly adopted to understand and manage the broadening scope of public mental health services.

An unanticipated consequence of the introduction of new professional categories into mental health units has been an expansion of their responsibilities. This is illustrated by the experience of daily bed meetings in large tertiary hospitals in which 10 or more psychiatrists and members of their teams spend 30 minutes or more focused on the social barriers to discharge. Too often I have witnessed the impact that making one patient’s admission contingent on another patient’s discharge can have on clinical decision-making.

I am not arguing that safe and secure accommodation is not important for patient mental health, only that it is not an area of specific expertise of psychiatrists (or any medical specialist/generalist). If this task was shifted to other members of the team, or other divisions of the social welfare system, it could free up 5–10% of the time of public psychiatrists and reduce the workforce gap by a similar amount.

PAs raise a potentially controversial question regarding appropriate governance. In the standard model, a PA performs less complex tasks under physician supervision. However, this relationship may be different for PAs supervised by physicians or GPs than for psychiatrists. The former groups would be supervising PAs on less complex tasks that are part of their main practice; while for many public psychiatrists they would be supervising PAs on tasks that they rarely or never do. While it is controversial whether psychiatrists should be capable of providing medical care at the level of primary practice or not, I leave it to one side as peripheral to the current argument.

Even more than PAs, the introduction of new health roles such as Peer worker and First Nations Worker into public mental health teams implicitly makes psychiatrists responsible for oversight of roles in which they are unlikely to have any expertise.

Thus, while it has been administratively convenient for psychiatrists to remain as the single point of responsibility for public mental health teams, the mental health environment has changed so much that it is time for a re-examination of the core responsibilities of psychiatrists in the optimal workforce mix of psychiatry, allied health, NGO/self-regulated workforces,^5^ inpatient/outpatient, and public/private as a precondition for workforce planning.

One measure of the success of such a re-examination would be an organisational structure where psychiatrists had little to no role in areas outside their core expertise, such as securing accommodation. In those areas they would operate as true consultants, for example, by providing opinions on the risks and benefits of discharge, or recommendations regarding conditions, without routine involvement in accommodation logistics or decisions.

Interesting new approaches to medical workforce analysis can aid such a re-examination. Furst and colleagues argue that the primary psychiatric workforce question should not be ‘How do we increase the number of psychiatrists per capita’ but ‘Given reasonably available resources, what mix is likely to achieve the best outcomes for patients?’ By applying a standardised classification system to local-level data they avoid the ecological fallacy of basing workforce decisions on national indicators, allowing them to inform local decision-making where the actual allocation of staff and service occurs.^3^

## Conclusions

The expanding cast of health workers, as well as the expectation that public mental health units provide holistic care that attends to all aspects of patients’ cultural and socioeconomic circumstances, challenges the traditional model of care. Part of the reason there are not enough psychiatrists to cover current needs is that we have increasing responsibility for areas outside our core expertise, across a more diverse group of patients, with a broader set of diagnoses. While increasing the number of psychiatrists and other mental health workers may be part of the solution, there is a need to fundamentally reconsider the scope of practice of psychiatrists and the hierarchical model that makes them the single point of responsibility for decisions outside their control.
